# Effectiveness, cost-effectiveness and implementation of WHO psychological interventions for migrant populations resettled in Italy: study protocol for the ADVANCE hybrid type-I randomized controlled trial

**DOI:** 10.3389/fpubh.2026.1905759

**Published:** 2026-10-21

**Authors:** Marianna Purgato, Federico Tedeschi, Eleonora Prina, Giulia Turrini, Beatrice Compri, Andrea Chiccoli, Maria Marti-Castaner, Manuela De Allegri, Hoa Thi Nguyen, Sanne Feenstra, Sara Khalil, Ulrich Reininghaus, Leonie Fleck, Liuska Sanna, Maritta Välimäki, Maija Satamo, Evaldas Kazlauskas, Austeja Dumarkaite, Matthias Kliegel, Chiara Scarampi, Melanie Mack, Cláudia de Freitas, Joyce Anne Quinto, Riccardo Serra, Marit Sijbrandij, Prasansa Subba, Lorenzo Tarsitani, Wietse Anton Tol, Michela Nosè, Corrado Barbui

**Affiliations:** 1 WHO Collaborating Centre for Research and Training in Mental Health and Service Evaluation, Department of Neurosciences, Biomedicine and Movement Sciences, Section of Psychiatry, University of Verona, Verona, Italy; 2 Department of Public Health, University of Copenhagen, Copenhagen, Denmark; 3 Heidelberg Institute of Global Health, University Hospital and Faculty of Medicine, University of Heidelberg, Heidelberg, Germany; 4 Faculty of Behavioural and Movement Sciences VU, Amsterdam, Netherlands; 5 Department of Human Neurosciences, Sapienza University of Rome, Rome, Italy; 6 Department of Public Mental Health, Central Institute of Mental Health, Medical Faculty Mannheim, Heidelberg University, Mannheim, Germany; 7 Health Service and Population Research Department, Institute of Psychiatry, Psychology & Neuroscience, King's College London, London, United Kingdom; 8 ESRC Centre for Society and Mental Health, King's College London, London, United Kingdom; 9 German Center for Mental Health (DZPG), Partner Site Mannheim-Heidelberg-Ulm, Mannheim, Germany; 10 Mental Health Europe, Brussels, Belgium; 11 Department of Nursing Science, Faculty of Medicine, University of Turku, Turku, Finland; 12 Hong Kong Metropolitan University, Hong Kong SAR, China; 13 Center for Psychotraumatology, Institute of Psychology, Faculty of Philosophy, Vilnius University, Vilnius, Lithuania; 14 Centre for the Interdisciplinary Study of Gerontology and Vulnerability (CIGEV), University of Geneva, Geneva, Switzerland; 15 Swiss National Center of Competences in Research LIVES–Overcoming Vulnerability: Life Course Perspectives, Geneva, Switzerland; 16 Faculty of Psychology and Educational Sciences, University of Geneva, Geneva, Switzerland; 17 Center for Psychology at University of Porto (CPUP), Porto, Portugal; 18 Faculty of Psychology and Education Sciences – University of Porto, Porto, Portugal; 19 Department of Clinical, Neuro and Developmental Psychology, WHO Collaborating Center for Research and Dissemination of Psychological Interventions, Amsterdam Public Health Research Institute, Vrije Universiteit Amsterdam, Amsterdam, Netherlands

**Keywords:** cost-effectiveness, Doing What Matters in Times of Stress, implementation science, migrants, psychological interventions, randomized controlled trial, refugees, Self-Help Plus

## Abstract

**Introduction:**

Migrant populations are exposed to multiple psychosocial stressors throughout the migration process, increasing their risk for common mental health conditions. There is increasing evidence for psychological interventions amongst migrants, although intervention effects tend to attenuate over time and efficacy against placebo is unknown. At the same time, implementation science questions are becoming critical, such as the benefits of in-person and online psychological interventions.

**Methods and analysis:**

We present a study protocol for a randomized hybrid type-I effectiveness-implementation study that will enroll adult migrants resettled in Italy who experience elevated psychological distress. Participants (*n* = 237) will be randomized to receive either in-person Self-Help Plus (SH+) or online Doing What Matters in Times of Stress (DWM). We will assess the performance of both SH + and DWM with an additional booster session, and will compare them against a psychological placebo without active intervention ingredients. An intention-to-treat analysis using linear mixed models will be conducted to explore interventions’ effects on depressive and anxiety symptoms at 3 months following randomization as measured by the Patient Health Questionnaire-Anxiety and Depression Scale, and to explore a set of implementation outcomes. We will also measure depressive symptoms, anxiety symptoms, mental health status, functioning, wellbeing, cost associated with care, quality of life, and self-stigma as secondary outcomes.

**Ethics and dissemination:**

The study protocol, informed consent procedures, and data protection measures were approved by the Comitato Etico Territoriale (CET) Area Sud Ovest Veneto (Approval ID n. 68505). The results of the present trial will be disseminated through the ADVANCE policy and scaling up strategy, in peer-reviewed journals, and at international and national scientific meetings.

**Trial registration number:**

ClinicalTrials.gov, identifier: NCT07209306.

## 1 Introduction

Migrant populations represent a substantial and growing proportion of the global population. The term “migrant” refers to a wide range of population groups, such as labor migrants, undocumented migrants, asylum seekers, refugees, internally displaced persons, and other people on the move (1). According to the latest International Organization for Migration (IOM) migration data, the number of international migrants worldwide has continued to grow. By mid-2024, an estimated 304 million people were living outside their country of birth or citizenship, reflecting continued global mobility and demographic shifts. This represents an estimated 3.8% of the world’s population (2).

Migration to Europe remains a significant element of global migration. Although specific IOM figures for Italy arrivals through 2024–2025 vary by route and source, recent reports suggest that tens of thousands of migrants continue to arrive in Europe via Mediterranean routes, with Italy being one of the principal entry points. For example, according to estimates published in news and migration analyses, over 60,000 people reached Italy in 2024 via irregular routes from North Africa (3).

Migrant populations are exposed to various risk factors for mental health conditions, such as discrepancies between pre-migration expectations and actual post-migration conditions, inadequate support systems, discrimination, challenges in adaptation and acculturation processes, as well as financial, administrative, and legal hurdles. Such stressors often impede their daily functioning and lives during and after the migration process. Among forcibly displaced populations such as refugees and asylum seekers, the additional adversities of loss, violence, and persecution further elevate vulnerability to mental disorders. As a consequence of this cumulative exposure to psychosocial stressors, mental health conditions are more frequent among migrants compared with the general population (4–8).

The World Health Organization (WHO) has developed a number of potentially scalable psychological interventions for populations affected by adversity (9). These include Self Help Plus (SH+) and its book and online version Doing What Matters in Times of Stress (DWM) (9). In this trial DWM will be evaluated as a standalone guided online intervention. A core feature of WHO psychological interventions is that they can be delivered by non-specialist helpers, such as trained peers or psychosocial workers, following appropriate training and supervision, an approach commonly referred to as task sharing. These interventions have been designed to be broadly applicable to individuals with or without a diagnosed mental health condition and to address a range of mental health problems in a transdiagnostic manner, including depression, anxiety, and post-traumatic stress disorder (PTSD) symptoms, while remaining easily adaptable to different populations, cultures, and languages. Additionally, the interventions and their implementation materials are available open access on the WHO website (10).

SH+ and DWM are grounded in principles of acceptance and commitment therapy (ACT) and aim to strengthen stress management and coping skills that can be flexibly applied across a wide range of emotional and psychosocial difficulties. Despite their shared theoretical foundation and objectives, SH + and DWM differ in their delivery modes and the level of interpersonal support they provide. SH + is a group-based, face-to-face intervention, typically delivered to large groups of up to 30 participants. Sessions are guided by one facilitator and a co-facilitator who play a supportive and organizational role, ensuring fidelity to the intervention. The content is largely delivered through standardized audio recordings and accompanying materials, allowing for consistent implementation across diverse settings and populations. DWM, on the other hand, represents a book version of the same core principles, which has been subsequently further adapted for online delivery. The intervention is typically accessed individually via digital platforms and is guided by a helper, who provides brief telephone or message-based support support following a protocol developed by WHO, to encourage engagement and completion, rather than intensive therapeutic input (11). This format allows DWM to be implemented in contexts where face-to-face delivery is not feasible, such as during public health emergencies or in settings with limited access to trained facilitators.

Both interventions (SH+ and DWM) are supported by evidence of beneficial effects. SH + has been evaluated among diverse populations of asylum seekers and refugees in Europe, Turkey, and northern Uganda (12–16). DWM has been examined in multiple randomized controlled trials (RCTs), including three studies conducted in Europe among vulnerable populations, such as migrants and individuals experiencing social adversity. In addition, DWM was tested in an RCT involving health workers in Spain as part of the RESPOND project (17, 18) and in a separate RCT with health workers in Italy during the COVID-19 pandemic (19, 20).

In addition to evaluating effectiveness on symptom outcomes, there is a growing recognition of the importance of examining implementation outcomes when assessing scalable psychological interventions. Understanding factors such as feasibility, acceptability, appropriateness, fidelity, and reach is essential to determine whether these interventions can be successfully delivered in real-world settings and sustained over time. This is particularly relevant for low-intensity, task-sharing approaches such as SH+ and DWM, which are specifically designed for large-scale dissemination across diverse and resource-constrained contexts. Hybrid effectiveness-implementation trial designs offer a valuable methodological framework to simultaneously assess clinical outcomes and implementation processes, thereby enhancing the relevance and applicability of findings for policy and practice.

In addition to these implementation research goals, the current study aims to fill two remaining gaps in knowledge on the efficacy of SH+ and DWM. First, although data from RCTs indicate that SH+ and DWM are effective, their effects appear to attenuate over the medium to long term (12–15). One potential strategy to address this decline in efficacy may be the incorporation of booster sessions, which may reinforce key therapeutic principles and skills after completion of the core intervention (19, 20). Booster sessions may help consolidate learning, promote continued practice of adaptive coping strategies, and support the maintenance of treatment gains over time, particularly in populations exposed to ongoing stressors (21–25). In the context of scalable, low-intensity interventions such as SH+ and DWM, booster sessions could represent a feasible and pragmatic approach to enhancing long-term effectiveness, particularly when integrated into stepped-care or task-shifting models of mental health service delivery.

A second concern with the existing evidence is that both SH+ and DWM have been evaluated primarily against usual care. To date, the only study employing a control condition comprising an equally supervised and structured alternative activity was conducted by Riello and colleagues, in an RCT among workers in residential nursing and care homes (18). In this study, DWM was delivered online under supervised conditions with brief support of a trained facilitator. At post-intervention, the preregistered analyses revealed no significant differences in self-reported anxiety or post-traumatic symptomatology between participants receiving SH+ and those engaged in an alternative activity. These findings raise the question of whether the effectiveness of SH+ may be largely attributable to nonspecific factors associated with participation in a psychological intervention, rather than to intervention-specific mechanisms (18). Apart from this study, no other trials have compared SH+ or DWM with a control condition providing a comparable number of sessions and level of support but without active intervention ingredients; instead, most studies have relied on usual care comparators. This limits the ability to distinguish between intervention-specific effects and nonspecific therapeutic factors.

## 2 Objectives

This hybrid type-I effectiveness-implementation RCT aims to examine the effectiveness and implementation outcomes of online (DWM) and in-person-delivered psychological interventions (SH+) compared with a psychological placebo arm, in reducing symptoms of anxiety and depression among migrants experiencing elevated psychological distress. Key implementation outcomes include the feasibility of delivery and the potential for scaling up interventions. Both interventions will have a digital booster session to examine strengthening the sustainability of intervention benefits.

The following three intervention strategies will be compared:

1 A contextually adapted version of SH+, delivered in groups of up to 30 people, in person, and complemented with a booster session;
2 A contextually adapted version of DWM, delivered as a guided online individual intervention, and complemented with a booster session;
3 A psychological placebo condition, administered individually with the same frequency and dosage of the WHO psychological interventions, and consisting of phone calls (or online meetings) with a helper, and complemented with an additional session.

We aim to conduct a comprehensive economic evaluation alongside this RCT to assess the economic costs of implementing the two interventions and the psychological placebo, estimate their cost-effectiveness and extended cost-effectiveness relative to the psychological placebo condition, and estimate the budget impact of the most probable upscaling scenarios.

## 3 Methods and analysis

### 3.1 Design

The study is part of an EU-funded project named “Addressing mental health vulnerabilities from adolescence to older age: innovating prevention science for times of change” (ADVANCE, no. 101080323)^1^. The present study is a hybrid type-I effectiveness-implementation RCT comparing different delivery modalities of WHO psychological interventions with a psychological placebo. The study will randomize adult migrants resettled in Italy who experience elevated psychological distress to one of three intervention arms: SH+ with a booster session, DWM with a booster session, or a psychological placebo with an additional session. Considering that all participants have a migration background, prior to randomization, those eligible for inclusion will receive a WHO intervention called Psychological First Aid (PFA), consisting of a brief, one-session intervention designed to reduce initial distress and promote adaptive functioning. All participants will also receive enhanced care as usual (ECAU), including informational materials (e.g., flyers) on locally available referral options and social and legal services for migrant populations resettled in Italy. The primary endpoint will be the change in symptoms of depression and anxiety from baseline to 3 months after randomization.

An Ethics and Data Advisory Board (EDAB) will monitor and provide expert advice on ethical, legal and societal issues that arise within the project. The study results will be reported in accordance with the Consolidated Standards of Reporting Trials statement (26).

To inform the development of this protocol and as a preliminary step, we conducted a separate mixed-methods co-creation study to foster a partnership among local research teams, representatives of the target population, and various stakeholders (i.e., clinical psychologists, social workers, representatives of NGOs, end-users) (27). This resulted in the establishment of a Society Advisory Group (SAG) at the onset of the project who will partner with the research team throughout its lifecycle to co-develop, culturally and contextually adapt, evaluate, and disseminate strategies for implementing and scaling up psychosocial interventions. The main changes that we integrated in this protocol after the SAG consultations are related to: the definition of the vulnerable groups composing the target population, recruitment strategies and implementation scenarios, the choice of outcome measures (i.e., stigma and implementation). The protocol of the separate mixed-methods study was approved by Comitato di Approvazione della Ricerca sulla Persona – CARP – of the University of Verona (n. 35/R1/2023, 06.02.2024).

### 3.2 Study participants

Investigators will proactively approach local organizations providing social, health, and/or legal support to migrant populations resettled in Verona and Rome, including refugees and asylum seekers, to identify potentially eligible participants. Specifically, participants will be recruited through (a) governmental and non-governmental organizations (NGOs); (b) targeted social media recruitment and word of mouth; and (c) other community-based organizations offering legal, social, administrative, psychological, or religious support to this vulnerable group.

Participants will be eligible to participate in the study if they meet the following criteria:

- 18 years or older;
- Migrant resettled in Italy temporarily or permanently (including migrant workers, undocumented migrants, asylum seekers, refugees, or other persons on the move);
- Elevated levels of psychological distress (Kessler Psychological Distress Scale (K10) > 15.9) (28);
- Sufficient mastery (written and spoken) of one of the languages the SH+/DWM intervention is being delivered in (i.e., English, Italian);
- Oral and written informed consent before entering the study.
- Potential participants who meet the inclusion criteria will be excluded from participation in this study if they meet any of the following criteria:
- Planning to permanently move back to their home country before the last follow-up assessment;
- Having acute medical conditions (requiring hospitalization);
- Imminent suicide risk, or expressed acute needs or protection risks that require immediate follow-up based on ad hoc questions and clinical judgement of a clinical psychologist;
- Having a severe mental disorder (e.g., psychotic disorders, substance-dependence) based on ad hoc questions and clinical judgement of a clinical psychologist;
- Having severe cognitive impairment based on ad hoc questions and clinical judgement of a clinical psychologist (e.g., severe intellectual disability or dementia);
- Currently receiving specialized psychological treatment (e.g., Cognitive Behavioral treatment (CBT), Eye Movement Desensitizing and Reprocessing (EMDR));
- In case of current psychotropic medication use: not being on a stable dose for at least 2 months.

Study participants will undergo four assessments: T0 (screening for eligibility, including assessment of psychological distress); T1 (baseline assessment); T2 (1 week after the booster session, 3 months after randomization); and T3 (3 months after the booster session, 6 months after randomization).

### 3.3 Informed consent procedure and screening

Before being enrolled in the study, interested individuals will be informed in plain language about the nature and scope of the study, potential risks and benefits, voluntary nature of participation, confidentiality, and the right to withdraw at any time without providing explanations and without any consequences. Participation will be completely voluntary to prevent biased responding. Participants who agree to participate will be asked to complete a written consent form.

After signing the informed consent form, participants will be invited to the screening interview. Screening will consist of a set of (self-administered) measurement instruments to check whether participants meet the inclusion criteria (Table 1). Additionally, specific questions will be asked to determine whether participants should be excluded. We will inform participants of the reasons why they may or may not be included in the study. Participants meeting the eligibility criteria will be included in the study and randomized.

**Table 1:** Overview of the concepts, their measures, the type of study parameter in the study, and the moment of measuring during the study phase.

Concept	Measure	Type of parameter	Screening (T0)	Baseline (T1)	Random allocation	Post-intervention (T2)	Follow-up (T3)
Psychological distress	K10	Screener	X		X	X	X
Suicide risk	Ad-hoc question during clinical interview	Screener	X			X	X
Mental, neurological or substance use disorders	Ad-hoc question during clinical interview	Screener	X				
Severe cognitive impairment	Ad-hoc question during clinical interview	Screener	X				
Informed consent procedure	Ad-hoc form during clinical interview	Other	X				
Socio-demographics	Ad-hoc form during clinical interview	Other	X	X			
Depressive and anxiety symptoms	PHQ-ADS	Primary		X		X	X
Depressive symptoms	PHQ-9	Secondary		X		X	X
Anxiety symptoms	GAD-7	Secondary		X		X	X
Current mental health	GHQ-12	Secondary		X		X	X
Functioning	WHODAS	Secondary		X		X	X
Well-being	WHO-5	Secondary		X		X	X
Self-stigma	ISMI	Secondary		X		X	X
Costs associated with health (and other) related care	CSRI	Economic		X		X	X
Quality of Life	EQ-5D-5L	Economic		X		X	X
Losses to follow-up	Participants failing to complete the outcome assessments or who withdraw consent	Secondary, Implementation				X	X
Intervention fidelity	WHO forms, metadata	Implementation				X	
Intervention acceptability	Adverse events, No. of sessions accessed, AIM	Implementation				X	X
Intervention appropriateness	IAM	Implementation				X	X
Intervention feasibility	FIM	Implementation				X	X

### 3.4 Random allocation

Randomization will occur at the individual level and will be centrally coordinated by the WHO Collaborating Centre at the University of Verona. After the screening and the baseline assessment, participants will be randomized to either SH+, DWM or psychological placebo, with an equal probability of assignment to each group (allocation ratio 1:1:1). The randomization will be stratified by recruitment area (Verona or Rome). The randomization schedule will be generated using the web-based software REDCap (29), using a variable block randomization method, in order to allocate groups randomly permuted in blocks of unequal size. The site investigators will not know the block size and will not be able to access the randomization list. The randomization list will be accessible only to the data manager, who will not be involved in participant enrolment. Additionally, and to conceal the allocation sequence, REDCap will allow for random allocation only after the main information on the enrolled participant has been entered, following verification of the inclusion criteria. After random allocation, the software will produce a unique identification number for each participant.

### 3.5 Interventions

The delivery of SH+, DWM, and PFA will be facilitated by peer helpers with at least primary-level education (e.g., master students in psychology, cultural mediators, PhD students), as well as experience and skills in providing psychosocial support (Supplementary material). Helpers speak the same language as the participants (as well as being able to communicate in English). It is not necessary for helpers to have a psychosocial or mental health background in order to be able to offer these interventions. Necessary skills include foundational helping skills, such as effective community-building and rapport-building, as well as experience supporting individuals in distress. Helpers will receive training in delivering SH+, DWM, and PFA from trainers (who are registered clinical psychologists trained by the WHO), and continuous supervision throughout the study period (Supplementary material).

#### 3.5.1 Psychological first aid (PFA) (all participants)

Before randomization, all eligible participants will be offered individual PFA through a telephone conversation or teleconferencing meeting. PFA is a WHO-developed support strategy that provides humane, supportive, and practical assistance to individuals affected by severe humanitarian crises. PFA focuses on five basic elements that are crucial to promote in the aftermath of crises, i.e., a sense of safety, calm, self-efficacy, community efficacy, connectedness, and hope (30). PFA consists of a (telephone) conversation (30–45 min) that a helper has with a participant. The helper provides non-intruding practical care and support, assesses needs and concerns, helps people to address basic needs, listens to people without pressuring them to talk, comforts people and helps them to feel calm (29).

#### 3.5.2 Enhanced care-as-usual (all participants)

In addition to PFA, all participants will receive ECAU. ECAU will include an information flyer outlining locally available services for migrants, including educational, health, social, and legal support. The flyer may also include information on community-based care, social and legal assistance, and psychoeducation. The flyer will also provide an overview of how the Italian healthcare system operates, and information on locally available referral options. For all participants, we will record whether they have a general practitioner, subject to their consent. If consent is provided, the general practitioner’s name and contact details will also be recorded.

#### 3.5.3 Self help plus

The SH+ intervention, developed by WHO, is a five-session stress management course (31) designed to be relevant for large segments of adversity-affected populations: it is intended to be transdiagnostic, easily adaptable to different cultures and languages and both meaningful and safe for people with and without mental disorders.

Based on ACT, SH+ emphasizes the concept that ongoing attempts to suppress unwanted thoughts and feelings can paradoxically make these problems worse. Instead, it highlights learning new ways to accommodate difficult thoughts and feelings, while guiding people to take proactive steps towards living in a way that is consistent with their values. ACT has been shown to be useful for a range of mental health issues and has been used successfully in a guided self-help format (30, 31). Therefore, SH + includes ACT techniques that aim to help people cope with stress, respond compassionately to themselves and others and to live in accordance with their values.

SH+ has two components: a pre-recorded course and an illustrated self-help book (DWM) (see Appendix). Pre-recorded audio material is delivered across five 90-min sessions (or modules) to groups of up to 30 people. During the sessions, that are delivered in person, participants have the opportunity to learn and practice SH+ techniques and to engage in brief discussions on specific topics according to the intervention manual. These discussions are structured and guided by the standardized SH+ manual. Facilitators support the delivery of the sessions and participants’ engagement but cannot introduce, modify, or elaborate on content beyond that specified in the manual.

We have adopted the English SH+ manual adapted for individuals with a migration background within the REDEFINE project (Grant No. 779255) (13). In ADVANCE, the SH + manual was translated into Italian for migrant populations in accordance with the WHO protocol (31).

The booster session of SH+ will be provided individually to participants in an online format (Supplementary material). The session will last approximately 20 min and include a summary of the five sessions. The booster session has been developed in collaboration with WHO.

We will assess SH+ protocol adherence using WHO ad-hoc forms completed by the facilitators. Additionally, the intervention supervisor (a clinical psychologist or psychiatrist) will observe at least 10% of the sessions and complete an adherence form.

#### 3.5.4 Doing what matters in times of stress

DWM (also known as the SH + book) contains the same content as SH+, training users in the five basic stress management skills through standalone audio. Additionally, participants are guided by a briefly trained helper.

In this study, DWM will be delivered individually as an online guided intervention. The DWM intervention has been adapted for use on a smartphone or other device. The DWM format is innovative in that it aims to ensure key intervention components are delivered as intended using pre-recorded audio. A new DWM module (i.e., section) is released every week. Therefore, participants will be asked to complete the entire DWM intervention within 5 weeks, with guidance from a helper through brief weekly phone or text-based contacts of approximately 15 min following a protocol devised by WHO and tested in a number of RCTs with different self-help interventions (32), including previous studies of DWM in the RESPOND project (14, 17).

We will adopt the DWM manuals in Italian and English, adapted for migrant populations within the RESPOND project (14). We will adopt the online DWM platform developed by the Vrije Universiteit Amsterdam.

The DWM booster session will be provided individually in an online format. It has been developed in collaboration with the WHO and contains a summary of the online sessions. The booster session will be facilitated by a trained helper.

We will assess DWM protocol adherence at the post-intervention stage using metadata collected during the intervention, including tracking participants’ use of the DWM app (e.g., which pages they access, how often, and how long they spend on the app). We will use this metadata only during the intervention to remind each participant (e.g., via email) after they complete each module that they should proceed to the next module. Moreover, participants will receive a weekly phone call from a helper to check on their progress and assess their well-being. Finally, the intervention supervisor (a clinical psychologist or a psychiatrist) will observe at least 10% of the phone contacts.

#### 3.5.5 Psychological placebo

The psychological placebo will consist of five phone calls (or online meetings) based on a script delivered by a helper, of the same duration as the DWM sessions (Supplementary material). In the first part of the call, participants will be reminded of locally available social, health, legal, and educational services. In the second part, participants will select a discussion topic from a list prepared by the research team (33).

The psychological placebo will be supplemented with an additional online individual session to mirror the booster sessions of SH+ and DWM, with same duration but with neutral content.

### 3.6 Primary and secondary outcomes

The primary outcome will be the change in symptoms of depression and anxiety from baseline to 3 months after randomization (T2), measured with the Patient Health Questionnaire–Anxiety and Depression Scale (PHQ-ADS) scale (34) administered by a masked trained assessor (Table 1).

The primary outcome will be evaluated using a standardized procedure, whereby assessors will read each item verbatim and record participants’ responses without clinical interpretation or modification, preserving the participant-reported nature of the instrument. This procedure will be applied consistently across both study sites. Interviewer administration was selected only for the primary outcome because it represents the main endpoint of the trial, and it allows a standardized administration procedure, promotes data completeness, and minimizes bias risk and variability due to misunderstanding of questionnaire items (35, 36).

Secondary outcomes include the following measures: a composite measure of depression and anxiety (PHQ-ADS) at T3 (34); depressive symptoms (PHQ-9) at T2 and T3 (37); anxiety symptoms (GAD-7) at T2 and T3 (38); current mental health (General Health Questionnaire-12) at T2 and T3 (39); functioning (WHODAS) at T2 and T3 (40); wellbeing (WHO-5) at T2 and T3 (41); self-stigma (Internalized Stigma of Mental Illness – ISMI) at T2 and T3 (42); use of care services and associated costs [the adapted European version of the Client Service Receipt Inventory (CSRI) questionnaire] at T2 and T3 (43); health related quality of life (EQ-5D-5L tool) at T2 and T3 (44); losses to follow-up at T2 and T3; intervention fidelity (WHO forms and metadata) at T2; intervention acceptability (adverse events, n. of sessions accessed and Acceptability of Intervention Measure – AIM at T2 and T3) (45); intervention appropriateness (Intervention Appropriateness Measure – IAM) (45) at T2 and T3, intervention feasibility (Feasibility of Intervention Measure – FIM) (45) at T2 and T3. Adoption and penetration will be assessed during the process evaluation phase.

### 3.7 Description of the outcome measurement tools

#### 3.7.1 Screener and socio-demographic information

##### 3.7.1.1 Psychological distress

Psychological distress will be measured using the K10 (28), a 10-item self-report questionnaire to screen for psychological distress in the past 30 days. Items are rated on a five-point Likert scale, with a total score from 10 to 50. The K10 has strong psychometric properties and exhibits strong discriminatory power in distinguishing DSM-IV cases from non-cases (28, 46). Moreover, the K10 has been found to have no substantial bias by education level or gender, making it a useful tool for research (47). The K10 is available in various languages, including English and Italian.

##### 3.7.1.2 Socio-demographic information

Socio-demographic information will be collected with predefined items based on the REDEFINE, RESPOND and STRENGTHS studies (13, 14, 48) (i.e., age, gender, nationality, years of education, relationship status, and employment status) and additional questions regarding country of birth, housing, household composition, household income on average, occupational area, mental health condition and overall current health status.

#### 3.7.2 Symptom outcomes

##### 3.7.2.1 Depressive and anxiety symptoms (psychological distress)

The PHQ-ADS is a 16-item primarily self-reported instrument that combines the nine-item Patient Health Questionnaire depression scale (PHQ-9) and the seven-item Generalized Anxiety Disorder scale (GAD-7) into a composite measure of depression and anxiety (36). The tool can be either self-administered or administered by an assessor, such as a clinical psychologist. In this RCT, the primary outcome will be assessed by a masked clinical psychologist. The score ranges from 0 to 48, with higher scores indicating higher levels of depression and anxiety symptoms (36). The evidence indicates high internal reliability (Cronbach’s alpha ranging from 0.8 to 0.9), strong convergent and construct validity, adequate unidimensionality and evidence of sensitivity to change. We will also analyze depressive and anxiety symptoms from the PHQ-9 and GAD-7 independently, as explained below.

##### 3.7.2.2 Depressive symptoms

Depressive symptoms during the past 2 weeks will be measured using the PHQ-9. It asks how often someone was bothered by each of the nine DSM-5 criteria and scores answers on a four-point Likert scale ranging from 0 (not at all) to 3 (nearly every day) (37). In addition to the nine items, the PHQ-9 asks: “If you checked off any problems, how difficult have these problems made it for you to do your work, take care of things at home, or get along with other people?,” which is to be answered with “Not difficult at all,” “Somewhat difficult,” “Very difficult,” or “Extremely difficult” (37).

##### 3.7.2.3 Anxiety symptoms

Anxiety symptoms will be assessed with the GAD-7, consisting of seven items that investigate the level of anxiety and worry (38). Each item is scored on a 0–3 scale, with a total score ranging from 0 to 21. Higher scores indicate more anxiety symptoms during the past 2 weeks. The GAD-7 scale has shown good psychometric properties (38). Validated and official versions of the GAD-7 are available in both English and Italian and can be downloaded from the PHQ website^2^.

##### 3.7.2.4 Mental health status

The 12-item General Health Questionnaire (GHQ-12) is a measure of current mental health (39). The questionnaire asks whether the respondent has experienced a particular symptom or behavior recently. Each item is rated on a four-point Likert scale, resulting in a total score of 36 or 12, depending on the GHQ version and the selected scoring method. The most common scoring methods are bi-modal (0-0-1-1) and Likert scoring styles (0-1-2-3).

##### 3.7.2.5 Functioning

Functional impairment will be assessed with the WHO Disability Assessment Schedule 2.0 (WHODAS) (40, 49). The WHODAS assesses health and disability. It is used across all diseases, including mental, neurological and substance use disorders. WHODAS covers six domains (cognition, mobility, self-care, getting along, life activities, and participation). It assesses the difficulties people have experienced across these domains over the last 30 days. We will use the 12-item version. Socio-demographic data (sex, age, education, marital status and work status) will be collected through questions A1-A5 of the WHODAS, which will be administered first (40, 49).

##### 3.7.2.6 Subjective wellbeing

Subjective wellbeing will be assessed using the WHO-5 Well-being Index (WHO-5), a 5-item questionnaire that measures current psychological well-being and quality of life, rather than psychopathology (41, 50). Scores range from 0 to 25. The scale has demonstrated sensitivity to change in well-being.

##### 3.7.2.7 Self-stigma

We will assess internalized stigma with the 29-item Internalized Stigma of Mental Illness (ISMI-29) (42). Each item is rated on a 4-point Likert scale ranging from 1 = strongly agree to 4 = strongly disagree. Total scores (calculated as the item means) range from 1 to 4, with higher scores indicating higher levels of internalized stigma. For descriptive purposes, a two-category cut-point method will also be applied, with mean scores up to 2.50 indicating lower internalized stigma versus scores above 2.50 indicating higher internalized stigma.

##### 3.7.2.8 Health-related quality of life

The intervention effects on health-related quality of life, measured as Quality-Adjusted Life Years (QALYs), will rely on the use of the EQ-5D-5L version, which consists of two sections: the EQ-5D descriptive system and the EQ visual analogue scale (EQ VAS) (44, 51). The descriptive system encompasses five dimensions: mobility, self-care, usual activities, pain/discomfort and anxiety/depression. Each dimension presents five levels of severity: no problems, slight problems, moderate problems, severe problems and extreme problems. Participants are prompted to indicate their health state by selecting the most fitting statement for each dimension. This selection yields a one-digit number representing the chosen level for that dimension. These digits for the five dimensions can be combined into a 5-digit number that characterizes the patient’s health state. The 5-digit number response will be combined with the estimated value sets for Italy to calculate the QALY scores for study participants at each assessment round.

The EQ VAS records the patient’s self-rated overall health on a vertical visual analogue scale, where a person marks their current health on a scale from 0 (worst imaginable health) to 100 (best imaginable health). The VAS complements the EQ-5D descriptive system by providing a subjective measure of overall health.

#### 3.7.3 Implementation outcomes

Implementation outcomes have been selected and will be interpreted according to Proctor et al.’s Implementation Outcomes Framework. Specifically, in addition to clinical effectiveness outcomes, the study will assess fidelity, acceptability, appropriateness, feasibility, and fidelity using the measures and procedures described below (52). This approach will inform future scaling-up of intervention strategies.

##### 3.7.3.1 Losses to follow-up

Losses to follow-up refer to participants who do not complete outcome assessments by the end of the study and participants who withdraw their consent during the study. The number of losses to follow-up and the reasons for attrition at T2 and T3 will be documented using ad hoc forms.

##### 3.7.3.2 Intervention fidelity

To determine whether the implemented intervention differs from the designed intervention, the research team will complete WHO fidelity forms. For DWM, we plan to use DWM metadata to track participants’ access to the intervention sessions.

##### 3.7.3.3 Intervention acceptability

The acceptability of SH+, DWM, and psychological placebo will be evaluated by collecting: (1) adverse events as reported by participants at T2 and T3; (2) the number of sessions accessed for each condition at T2 and T3; (3) scores on the Acceptability of Intervention Measure (AIM) subscale at T2 and T3.

Adverse events are defined as any undesirable experience occurring to a subject during the study, whether considered related to the trial procedure or the SH+ / DWM intervention. All adverse events reported spontaneously by the subject or observed by the investigator or staff will be recorded for ethical reasons and as a proxy measure of the intervention’s acceptability.

Number of sessions accessed for each condition: SH+ (number of group sessions attended), DWM (number of modules unlocked and number of helper calls) and psychological placebo (number of helper calls).

The Acceptability of Intervention Measure (AIM) is a subscale of (4 items, 5-point Likert scale: 1 = completely disagree to 5 = completely agree) (45). The unit of measure is a descriptive scale rating the level of acceptability of the intervention.

##### 3.7.3.4 Intervention appropriateness

The intervention appropriateness will be assessed at T2 and T3 with the “Intervention Appropriateness Measure” (IAM) subscale (4 item, 5-point Likert scale: 1 = completely disagree to 5 = completely agree) (45). The unit of measure is a descriptive scale that rates the appropriateness of the intervention.

##### 3.7.3.5 Intervention feasibility

The intervention feasibility will be assessed at T2 and T3 with the “Feasibility of Intervention Measure” (FIM) subscale (4 items, 5-point Likert scale: 1 = completely disagree to 5 = completely agree) (45). The unit of measure is a descriptive scale rating the level of feasibility of the intervention.

#### 3.7.4 Economic outcomes

##### 3.7.4.1 Use of health care and other care services and associated costs

The Client Service Receipt Inventory (CSRI) was developed for the collection of data on care service utilization (e.g., use of health care services, social care, time out of employment and other usual activities, need for informal care) and related socio-demographic characteristics of study participants, as the basis for calculating the costs of health care and other services. It has been used cross-culturally and is available for Italy (43). Since care service delivery systems differ across countries, it is essential to adapt the CSRI locally to ensure its appropriateness for use in the study population and settings. The existing previously adapted CSRI version for the migrant population in Italy will provide the core service items for a new adaptation to capture additional information useful for the extended cost-effectiveness analysis. Furthermore, detailed information regarding the direct payments/out-of-pocket expenses on service utilization and productivity loss due to mental ill health will be monitored by the adapted CSRI questionnaire to enable the generation of both cost-effectiveness and extended cost-effectiveness analyses.

### 3.8 Masking

Masking participants, helpers and research staff is not feasible due to the nature of the interventions. However, the trained assessor who will evaluate the primary outcome will be masked to intervention allocation. To assess the integrity of masking, assessors will be asked to guess the participant’s allocation following each assessment. At the beginning of each primary outcome assessment, the masked assessor will remind participants not to disclose their treatment allocation or discuss the intervention they received, in order to minimize the risk of accidental unmasking. In addition, the statistician performing the analyses will be masked to the treatment identity until completion of the primary analyses. Secondary outcomes will be collected through self-administered questionnaires using REDCap (28).

### 3.9 Sample size calculation

Based on findings from previous SH+ studies, such as those conducted with asylum seekers, refugees, and migrant population groups within the REDEFINE and RESPOND projects (13, 17), and the RCTs conducted by Tol with refugee women in Uganda (13, 17), a Cohen’s *f* effect size of 0.34 is hypothesized for the time-by-treatment interaction at 3 months post-randomization, comparing each active intervention arm (SH+ and DWM) with the psychological placebo arm on the primary outcome (PHQ-ADS) (36). Moreover, meta-analytic evidence on psychosocial and psychological interventions for refugees and asylum seekers indicates overall post-intervention effect sizes ranging from Hedges’ *g* = 0.82 to 1.05 across depression and anxiety outcomes, which correspond to Cohen’s *f* values between 0.41 and 0.53. To adopt a conservative estimate for our trial comparing active arms (SH + with booster sessions, DWM with booster sessions) against ECAU, we hypothesized an effect size of *f* = 0.34 for the time-by-treatment interaction (53, 54). To account for multiplicity, we will apply the Benjamini–Hochberg procedure. For the purpose of power calculations, a conservative significance threshold of *p* = 0.025 was assumed for each pairwise comparison in the within–between ANOVA interaction. This ensures adequate power to detect a significant effect in each intervention arm, even when the other intervention fails to reach significance. Under this assumption, 55 participants per study arm are required to achieve 90% power. Allowing for an anticipated attrition rate of 30%, 79 participants will be recruited per arm, resulting in a three-arm randomized controlled trial with a total sample size of 237 participants. Given the hybrid effectiveness-implementation design of the trial, a power calculation was also performed for implementation outcomes, based on the pre-attrition sample size of 55 participants per arm already determined by the primary outcome. Given that these outcomes are not assessed at baseline, no baseline adjustment will be possible, which reduces the statistical power compared to the primary outcome analysis. Should ceiling effects lead to dichotomization of these outcomes, and based on the conservative significance threshold of *p* = 0.025 discussed above, a two-sided *Z*-test for two proportions (pooled variance) would achieve at least 80% power to detect a difference between each intervention arm (SH+, DWM) and the psychological placebo, assuming a proportion of participants reaching a clinically meaningful target score of 90% in the active arm and 65% in the control arm (achieved power: 82.7%).

### 3.10 Statistical analysis

#### 3.10.1 Analysis of clinical and implementation outcomes

All primary and secondary analyses will be performed on an intention-to-treat (ITT) basis. The ITT population will consist of all participants randomly assigned to the competitive intervention strategies, for whom baseline assessment data are available. In order to check the robustness of results, the primary outcome will be additionally analyzed using a per-protocol (PP) approach, which will include only SH+ participants who completed at least three sessions, DWM participants clicking through all of the content of at least three modules (regardless of whether or not they engage in phone calls/messages with the helper) and psychological placebo participants engaging in at least three phone calls with the helper. The analysis of the PP population will be used solely for confirmatory purposes. If less than 5% of participants do not receive the allocated intervention according to the study protocol, the PP analysis will not be performed.

The primary outcome will be summarized using the number of subjects (n), means, and standard deviations (SDs). To compare the two intervention groups at baseline, standardized mean differences will be calculated. The primary analysis will simultaneously assess the treatment effect on the average PHQ-ADS score at each time point in the ITT population. The main conclusion of the trial will be based on the ITT analysis of the primary outcome (i.e., the effect on PHQ-ADS score at 3 months after random allocation). To estimate the intervention effect at the time-points T2 and T3, a linear mixed model will be employed for the PHQ-ADS analysis, with time as a fixed effect, baseline PHQ-ADS as a covariate, and subject as a random effect. The model will be re-parametrized by constraining the treatment fixed-effect for both intervention arms to be 0, and by including a time*treatment interaction at T2 as well. In this way, at each time-point, the treatment effects for both interventions will be measured as the interaction between time (as a categorical variable) and treatment. These interaction coefficients directly represent the mean difference between each intervention arm and the control arm at that specific time-point. To account for multiple testing (two intervention arms), the Benjamini–Hochberg procedure will be applied to the *p*-values of the primary outcome, that is the interaction at T2. We will use the model’s predicted values to estimate the average values if all participants were assigned to each arm. In addition, a covariate-adjusted mixed model of the primary endpoint will be performed by adding covariates showing imbalance at baseline (as measured by a standardized mean difference above 0.1 in absolute value). Robust standard errors will be used in all models.

A secondary analysis of the intervention’s effect on PHQ-ADS scores will be conducted using the PP population, following the same approach as reported above. In addition, a covariate-adjusted mixed model for the primary endpoint will be performed using the PP population, incorporating pre-specified baseline covariates (e.g., gender, age, education, marital status, and working condition, psychological symptoms at baseline).

Missing data at the scale level (that is, at least 50% of items missing) will be treated as missing at random (MAR). No imputations for missing values will be made, as multilevel models can handle missing data (55). In the case less than 50% of items are missing for a specific scale, we will perform the Corrected Item mean Substitution method (i.e., the item mean across participants weighted by the subject’s mean of completed items) (56), using information from subjects belonging to the same intervention arm for the same time-point (estimated values above the maximum or below the minimum admissible value will be set to maximum/minimum). As a sensitivity analysis, the analyses for outcomes with partially imputed scales were repeated by excluding such imputed values.

A linear mixed model with robust standard errors, as mentioned for the primary analysis, will be carried out to analyze the following secondary outcomes: psychological distress (GHQ-12), depressive symptoms (PHQ-9), anxiety symptoms (GAD-7), functioning (WHODAS), well-being (WHO-5), quality of life (EQ-5D-5L), and self-stigma (ISMI).

Intervention fidelity, acceptability, appropriateness, and feasibility will be analyzed as continuous or, in the case of ceiling effects, dichotomous outcomes, with a clinically meaningful (e.g., maximum score vs. other) cut-off chosen to ensure sufficient variability for the analysis.

The proportion of participants lost to follow-up will be compared between the three groups using a chi-square test or a Fisher’s exact test, as appropriate, depending on expected cell counts.

The secondary outcomes will be analyzed on the ITT population only.

To assess the plausible magnitude of within-group correlation, we examined data from a previous SH + trial (RE-DEFINE) for which facilitator/group membership information was available. Intraclass correlation coefficients (ICCs) for the group-level effect, estimated from repeated-measures mixed-effects models across five continuous outcome measures (general psychological distress, depression, PTSD symptoms, wellbeing, and disability), ranged from 0.06 to 0.14, indicating a modest but non-negligible degree of within-group correlation. Given the relatively small number of SH+ groups anticipated in ADVANCE, estimation of the between-group variance based on ADVANCE data alone would likely be imprecise. We will therefore conduct a sensitivity analysis for the primary continuous outcomes using a Bayesian hierarchical model that includes a random effect for SH+ group. The prior distribution for the between-group variance will be informed by the historical evidence from RE-DEFINE using a meta-analytic-predictive (MAP) approach (57, 58) adapted to the partially nested design of the trial (59, 60).

### 3.11 Analysis of economic outcomes

The economic evaluation will be conducted from a societal perspective and will comprise four components: (1) an activity-based micro-costing study, (2) a cost-effectiveness analysis (CEA), (3) a cost-utility analysis (CUA), and (4) an extended cost-effectiveness analysis (ECEA).

First, an activity-based bottom-up micro-costing study will be conducted to identify, measure, and value all costs associated with implementing the three interventions. Time-use data for researchers and implementing partners will be collected using pre-defined templates. Helper time will be valued using standardized unit costs reflecting the opportunity cost of the role performed, irrespective of whether helpers are fully remunerated, partially compensated, or contribute on a voluntary basis. Costing data will subsequently inform a budget impact analysis to estimate the costs of scaling up the interventions under alternative scenarios, in consultation with relevant stakeholders. We will also assess whether the interventions reduce healthcare and other care service costs, including participants’ out-of-pocket expenditures, following the intention-to-treat principle. Cost outcomes at T2 and T3 will be analyzed using generalized linear models with an identity link and gamma distribution, adjusting for baseline costs. Intervention effects will be tested using *z*-tests, with Benjamini–Hochberg correction for multiple testing.

Second, a CEA will be conducted using costs from the micro-costing study and the primary effectiveness outcome (PHQ-ADS score at 3 months post-randomization), following the same analytical procedures used for the primary trial outcome.

Third, a CUA will be performed using Quality-Adjusted Life Years (QALYs) derived from the EQ-5D-5L. Incremental cost-effectiveness ratios (ICERs) per QALY will be calculated for CUA and compared with country-specific cost-effectiveness thresholds. As Italy does not have an officially adopted cost-effectiveness threshold, results will be interpreted using cost-effectiveness acceptability curves across a range of willingness-to-pay thresholds, with particular reference to thresholds commonly adopted in the international literature. One-way and probabilistic sensitivity analyses will be used to assess parameter uncertainty. As Italy does not have an officially adopted cost-effectiveness threshold, results will be interpreted using cost-effectiveness acceptability curves over a willingness-to-pay range of €20,000–€40,000 per QALY, with results also reported at selected reference values of €25,000 and €40,000 per QALY. These values will be considered illustrative reference points used in the Italian health-economic literature rather than formally adopted national thresholds (61).

Finally, an extended cost-effectiveness analysis will estimate net intervention costs by accounting for financial risk protection through reduced out-of-pocket expenditures across socioeconomic groups. Together, the CEA, CUA, and ECEA will provide policymakers with comprehensive economic evidence complementing clinical findings to inform decisions on adoption and scale-up.

## 4 Ethics and dissemination

All data will be handled confidentially and coded using a unique code known only to the research team. Processing of personal data will comply with the General Data Protection Regulation (“GDPR”), as defined in Regulation (EU) 2016/679 of the European Parliament and of the Council of 27 April 2016, on the protection of individuals regarding the processing of personal data and the free movement of such data. Data will be stored in a locked record at the WHO Collaborating Centre of the University of Verona to ensure the confidentiality of the study participants. Only authorized research personnel will have access to this data. According to the ADVANCE Data Management Plan, the fully anonymized trial dataset will be made available through Zenodo, a European Commission-endorsed repository (62). Raw data containing personal or sensitive information will not be publicly shared and will be retained locally in accordance with national and institutional regulations. Each partner in ADVANCE is considered an independent controller, as defined in the GDPR, for its processing of personal data and will act in accordance with applicable data protection laws (including, but not limited to, the GDPR).

## 5 Discussion

The present study is expected to generate evidence with significant relevance for addressing psychological distress among migrant populations resettled in high-income countries. By adopting a hybrid effectiveness–implementation RCT design, the study will simultaneously evaluate effectiveness on symptom outcomes, implementation outcomes, and cost-effectiveness of WHO-psychological interventions in real-world settings.

Specifically, this trial will contribute to close gaps in the literature concerning the effectiveness of task shared psychological interventions for populations exposed to adversity. For the first time in this population, we will examine the effectiveness of SH+ and DWM compared with a psychological placebo delivered with the same frequency and dosage as the WHO interventions. Including a psychological placebo condition will allow disentangling the effects of intervention-specific mechanisms from nonspecific therapeutic factors. Such a design has not been employed previously in migrant populations (63). By contrast, a similar controlled condition among workers in residential nursing and care homes, where an alternative activity was provided, did not demonstrate any added benefit of the intervention (19). Clarifying whether observed effects are attributable to specific intervention components rather than nonspecific relational or contextual factors is therefore crucial.

Secondly, the inclusion of a digital booster session is expected to enhance the durability of treatment effects (21). By reinforcing core therapeutic principles and skills following the completion of the main intervention, the booster session can help consolidate learning, promote the continued use of adaptive coping strategies, and mitigate the attenuation of effects over time (64). This is particularly relevant for migrant populations, who are often exposed to persistent and cumulative stressors that may undermine longer-term intervention gains (65). If shown to be effective, the booster component would provide a pragmatic and scalable strategy to enhance the sustainability of WHO psychological interventions, with significant implications for their integration into stepped-care and task-shifting models, as well as for improving long-term mental health outcomes in resource-constrained and high-need settings.

From a public health perspective, this study addresses a critical gap in the evidence base for mental health interventions tailored to migrant populations, who are disproportionately exposed to psychosocial stressors and face substantial barriers to accessing specialized mental health care (8, 66). Demonstrating the effectiveness and implementation outcomes of low-intensity, non-specialist-delivered interventions may support their inclusion in early response strategies for newly arrived migrants, potentially mitigating the adverse mental health impact of post-migration stressors and preventing the escalation of distress over time (65, 67). The planned cost-effectiveness analysis will generate essential evidence on the value for money of these interventions, informing whether investment in their implementation and scale-up represents an efficient use of resources for health systems in Italy and other high-income resettlement contexts (68). Moreover, the budget impact analysis will generate evidence on the affordability of different scale-up scenarios. Importantly, the hybrid effectiveness–implementation design will produce actionable insights into the feasibility, acceptability, and integration of these WHO interventions within existing health, social, and legal service systems for migrants. Explicit attention to implementation outcomes is critical and innovative, as failures in uptake, fidelity, and sustainability – rather than solely intervention efficacy – are among the most common reasons evidence-based interventions do not achieve population-level impact (69, 70). Evidence on delivery modalities, participant engagement, and outcomes in routine care settings will support policymakers and service planners in selecting and adapting interventions that are not only clinically effective but also scalable and sustainable in real-world contexts. This study also has some limitations. First, even though the primary outcome will be evaluated by masked assessors, most clinical and implementation outcomes rely on self-reported measures, which may be subject to reporting and social desirability biases. Second, because of the nature of the interventions, participants and helpers cannot be masked to treatment allocation. This might therefore generate differential expectancy effects that cannot be fully controlled despite assessor masking and the inclusion of a psychological placebo condition. A further limitation is that for feasibility reasons eligibility will be restricted to participants with sufficient proficiency in English or Italian. Consequently, the findings may not be generalizable to recently arrived refugees who have not yet acquired basic language skills. However, this pragmatic choice was necessary to ensure feasible intervention delivery and follow-up while still targeting a population exposed to substantial post-migration stressors. Moreover, recruitment is restricted to Verona and Rome, which may limit the geographical and service-context generalizability of the findings. Finally, the six-month follow-up may be insufficient to establish whether the effects of the interventions and booster sessions are sustained over the medium and longer term.

Overall, the study is expected to contribute to the global evidence base guiding the implementation of WHO psychosocial interventions, support mental health policy and service planning for migrant populations, and advance the methodological application of hybrid trial designs in global and public mental health research.

## Patient and public involvement

Stakeholders have been involved since the early stages of the ADVANCE project through the establishment of a Society Advisory Group (SAG), comprising adults with a migrant background (end-users), mental health professionals, policymakers, and practitioners working in reception centers and community services (23). The SAG contributed to a mixed-methods co-creation study, and the insights generated informed the development of the present study protocol. Public representatives were not involved in the conduct of the trial or in outcome assessment. Patients were not involved in the design, or conduct, or reporting, or dissemination plans of this research.

## Footnotes

1
https://advancementalhealth.ku.dk/

2
www.phqscreeners.com
