# Supplementary material for: Effectiveness, cost-effectiveness and implementation of WHO psychological interventions for migrant populations resettled in Italy: study protocol for the ADVANCE hybrid type-I randomized controlled trial
Source: Front Public Health. 2026 Oct 21;14:1905759. doi: 10.3389/fpubh.2026.1905759 (PMC13730949; doi:10.3389/fpubh.2026.1905759)
Supplement: Supplementary file 1 [file Supplementary_file_1.docx]

**Effectiveness, cost-effectiveness and implementation of WHO psychological interventions for migrant populations resettled in Italy: study protocol for the ADVANCE hybrid randomised controlled trial**

**Supplementary material**

**Table of contents**

1. SPIRIT checklist
2. Glossary
3. Description of SH+
4. Description of the intervention Training (SH+, DWM, PFA)
5. Outline of the booster session
6. Outline of the psychological placebo session
7. Referral pathway for study participants in need of structured clinical support
8. Description of the implementation measures

**1. SPIRIT 2025 checklist of items to address in a randomized trial protocol***

| **Section / Topic** | **No** | **SPIRIT 2025 checklist item description** | **Reported on page no.** |
| --- | --- | --- | --- |
| **Administrative information** | | |  |
| Title and structured summary | 1a | Title stating the trial design, population, and interventions, with identification as a protocol | Reported |
|  | 1b | Structured summary of trial design and methods, including items from the World Health Organization Trial Registration Data Set | Reported |
| Protocol version | 2 | Version date and identifier | Reported |
| Roles and responsibilities | 3a | Names, affiliations, and roles of protocol contributors | Reported |
|  | 3b | Name and contact information for the trial sponsor | NA |
|  | 3c | Role of trial sponsor and funders in design, conduct, analysis, and reporting of trial; including any authority over these activities | Reported |
|  | 3d | Composition, roles, and responsibilities of the coordinating site, steering committee, endpoint adjudication committee, data management team, and other individuals or groups overseeing the trial, if applicable | Reported |
| **Open science** | | |  |
| Trial registration | 4 | Name of trial registry, identifying number (with URL), and date of registration. If not yet registered, name of intended registry | Reported |
| Protocol and statistical analysis plan | 5 | Where the trial protocol and statistical analysis plan can be accessed | Reported |
| Data sharing | 6 | Where and how the individual de-identified participant data (including data dictionary), statistical code, and any other materials will be accessible | Reported |
| Funding and conflicts of interest | 7a | Sources of funding and other support (e.g., supply of drugs) | Reported |
|  | 7b | Financial and other conflicts of interest for principal investigators and steering committee members | Reported |
| Dissemination policy | 8 | Plans to communicate trial results to participants, healthcare professionals, the public, and other relevant groups (e.g., reporting in trial registry, plain language summary, publication) | Reported |
| **Introduction** | | |  |
| Background and rationale | 9a | Scientific background and rationale, including summary of relevant studies (published and unpublished) examining benefits and harms for each intervention | Reported |
|  | 9b | Explanation for choice of comparator | Reported |
| Objectives | 10 | Specific objectives related to benefits and harms | Reported |
| **Methods: Patient and public involvement, trial design** | | |  |
| Patient and public involvement | 11 | Details of, or plans for, patient or public involvement in the design, conduct, and reporting of the trial | Reported |
| Trial design | 12 | Description of trial design including type of trial (e.g., parallel group, crossover), allocation ratio, and framework (e.g., superiority, equivalence, non-inferiority, exploratory) | Reported |
| **Methods: Participants, interventions, and outcomes** | | |  |
| Trial setting | 13 | Settings (e.g., community, hospital) and locations (e.g., countries, sites) where the trial will be conducted | Reported |
| Eligibility criteria | 14a | Eligibility criteria for participants | Reported |
|  | 14b | If applicable, eligibility criteria for sites and for individuals who will deliver the interventions (e.g., surgeons, physiotherapists) | Reported |
| Intervention and comparator | 15a | Intervention and comparator with sufficient details to allow replication including how, when, and by whom they will be administered. If relevant, where additional materials describing the intervention and comparator (e.g., intervention manual) can be accessed | Reported, Paper and Supplentary |
|  | 15b | Criteria for discontinuing or modifying allocated intervention/comparator for a trial participant (e.g., drug dose change in response to harms, participant request, or improving/worsening disease) | Reported |
|  | 15c | Strategies to improve adherence to intervention/comparator protocols, if applicable, and any procedures for monitoring adherence (e.g., drug tablet return, sessions attended) | Reported |
|  | 15d | Concomitant care that is permitted or prohibited during the trial | Reported |
| Outcomes | 16 | Primary and secondary outcomes, including the specific measurement variable (e.g., systolic blood pressure), analysis metric (e.g., change from baseline, final value, time to event), method of aggregation (e.g., median, proportion), and time point for each outcome | Reported |
| Harms | 17 | How harms are defined and will be assessed (e.g., systematically, non-systematically) | Reported |
| Participant timeline | 18 | Time schedule of enrollment, interventions (including any run-ins and washouts), assessments, and visits for participants. A schematic diagram is highly recommended (see Figure) | Reported |
| Sample size | 19 | How sample size was determined, including all assumptions supporting the sample size calculation | Reported |
| Recruitment | 20 | Strategies for achieving adequate participant enrollment to reach target sample size | Reported |
| **Methods: Assignment of interventions** | | |  |
| Randomization: |  |  |  |
| Sequence generation | 21a | Who will generate the random allocation sequence and the method used | Reported |
|  | 21b | Type of randomization (simple or restricted) and details of any factors for stratification. To reduce predictability of a random sequence, other details of any planned restriction (e.g., blocking) should be provided in a separate document that is unavailable to those who enroll participants or assign interventions | Reported |
| Allocation concealment  mechanism | 22 | Mechanism used to implement the random allocation sequence (e.g., central computer/telephone; sequentially numbered, opaque, sealed containers), describing any steps to conceal the sequence until interventions are assigned | Reported |
| Implementation | 23 | Whether the personnel who will enroll and those who will assign participants to the interventions will have access to the random allocation sequence | Reported |
| Blinding | 24a | Who will be blinded after assignment to interventions (e.g., participants, care providers, outcome assessors, data analysts) | Reported |
|  | 24b | If blinded, how blinding will be achieved and description of the similarity of interventions | Reported |
|  | 24c | If blinded, circumstances under which unblinding is permissible, and procedure for revealing a participant’s allocated intervention during the trial | Reported |
| **Methods: Data collection, management, and analysis** | | |  |
| Data collection methods | 25a | Plans for assessment and collection of trial data, including any related processes to promote data quality (e.g., duplicate measurements, training of assessors) and a description of trial instruments (e.g., questionnaires, laboratory tests) along with their reliability and validity, if known. Reference to where data collection forms can be accessed, if not in the protocol | Reported |
|  | 25b | Plans to promote participant retention and complete follow-up, including list of any outcome data to be collected for participants who discontinue or deviate from intervention protocols | Reported |
| Data management | 26 | Plans for data entry, coding, security, and storage, including any related processes to promote data quality (e.g., double data entry; range checks for data values). Reference to where details of data management procedures can be accessed, if not in the protocol | Reported |
| Statistical methods | 27a | Statistical methods used to compare groups for primary and secondary outcomes, including harms | Reported |
|  | 27b | Definition of who will be included in each analysis (e.g., all randomized participants), and in which group | Reported |
|  | 27c | How missing data will be handled in the analysis | Reported |
|  | 27d | Methods for any additional analyses (e.g., subgroup and sensitivity analyses) | Reported |
| **Methods: Monitoring** | | |  |
| Data monitoring committee | 28a | Composition of data monitoring committee (DMC); summary of its role and reporting structure; statement of whether it is independent from the sponsor and funder; conflicts of interest and reference to where further details about its charter can be found, if not in the protocol. Alternatively, an explanation of why a DMC is not needed | Reported |
|  | 28b | Explanation of any interim analyses and stopping guidelines, including who will have access to these interim results and make the final decision to terminate the trial | NA |
| Trial monitoring | 29 | Frequency and procedures for monitoring trial conduct. If there is no monitoring, give explanation | Reported |
| **Ethics** | | |  |
| Research ethics approval | 30 | Plans for seeking research ethics committee/institutional review board approval | Reported |
| Protocol amendments | 31 | Plans for communicating important protocol modifications to relevant parties | Reported |
| Consent or assent | 32a | Who will obtain informed consent or assent from potential trial participants or authorized proxies, and how | Reported |
|  | 32b | Additional consent provisions for collection and use of participant data and biological specimens in ancillary studies, if applicable | NA |
| Confidentiality | 33 | How personal information about potential and enrolled participants will be collected, shared, and maintained in order to protect confidentiality before, during, and after the trial | Reported |
| Ancillary and post-trial care | 34 | Provisions, if any, for ancillary and post-trial care, and for compensation to those who suffer harm from trial participation | NA |

**2. Glossary**

**SH+ facilitator**: A non-specialist provider trained to deliver the WHO Self-Help Plus (SH+) intervention. The SH+ facilitator (e.g., social or community worker, early career psychologist or master student in psychology) guides participants through the five sessions using the standardized audio recordings and written materials, according toWHO manuals. Their role is to create a safe and supportive environment, deliver the intervention protocol appropriately, facilitate group discussions, and manage practical aspects of session delivery. SH+ facilitators do not provide individualized psychological treatment, interpretation, or counselling, and do not adapt the intervention content beyond what is specified in the WHO manual.

**DWM helper**: A non-specialist provider trained to support the delivery of the WHO Doing What Matters in Times of Stress (DWM) intervention. The DWM helper (e.g., social or community worker, early career psychologist or master student in psychology) supports participants in engaging with the self-help materials and exercises, clarifies instructions when needed, and encourages the application of stress management skills in daily life. The helper’s role is limited to guidance and support; as for SH+ facilitators, helpers do not deliver psychotherapy, offer clinical advice, or tailor the intervention content. The DWM helper operates according to the standardized guidance and principles outlined by the WHO, reporting to supervisors about fidelity to the intervention and appropriate referral in case of risk or distress experienced by participants.

**Trainers/supervisors**

All SH+ facilitators and DWM helpers will be actively trained by trainers, who are registered (clinical) psychologists and will be supervised throughout the process. Trainers will also independently assess and monitor SH+ and DWM sessions at random to ensure treatment adherence and fidelity. Trainers will also supervise the assessments, the SH+ and DWM delivery, and the therapeutic process to reduce the burden on and risks for participants.

SH+ and DWM trainers/supervisors are five licensed mental health care professionals such as health-care psychologists or psychiatrists. They will be trained by a Master Trainer via a training-of-trainers (TOT) program, consisting of the same elements as the training for helpers, but also of training and supervision skills. Such training with continued supervision by professionals has been successfully used for lay-counsellors before.

The research team at the University of Verona (UNIVR) will be responsible for arranging the training and supervision for the facilitators and helpers. Supervision will take place on a weekly basis. This will be done remotely or face-to-face, depending on the preference of the trainers/supervisors. The trainers/supervisors will also receive supervision by the Master trainer when necessary.

**Master Trainers**

Master Trainers are experienced clinical psychologists based at UNIVR. They received training in SH+ and DWM by WHO technical officers, and have an extensive experience in delivering and supervising WHO-developed interventions.

**3. Description of SH+**

The SH+ sessions are as follows: Session 1: Grounding, which brings attention back to the present moment when caught up in distressing emotions. Session 2: Unhooking: Noticing difficult thoughts and feelings, naming them, and refocusing on what you are doing. Session 3: Acting on your values: Identifying personal values and then taking small or big actions to live in line with these values. Session 4: Being kind: Enhancing and encouraging kindness towards oneself and towards others. Session 5: Making room: Learning how to tolerate stress while still acting consistently with values.

The audio material conveys key information about stress management and guides participants through individual exercises and small-group discussions7. Written manuals help non-specialist facilitators to conduct the course using the pre-recorded audio.

**4. Description of intervention training**

**SH+ and DWM training**

Duration: five days per intervention – full time

The training is based on WHO manuals. The **Self-Help Plus (SH+) training** manual is for people or organizations wanting to train SH+ facilitators. It contains all the information needed to deliver a training and implement SH+. Part A covers key information for setting up and implementing SH+, including training and supervision requirements . Part B provides detailed instructions for the training workshop. Training manuals will be complemented by a set of slides provided by the WHO in the WHO website (https://who.canto.global/s/G2I3J?viewIndex=0).

The **DWM training** involves learning how to implement the 5-week program, focusing on the core intervention ingredients, such as mindfulness, relaxation, and behavioral changes.

Each facilitator/helper will receive a written manual as a guide for the brief support sessions. Additionally, DWM helpers will specifically practice delivering support remotely (i.e., practice providing support in role-play settings).

**Psychological First Aid (PFA)** **training**

Duration: half day/two days (depending on previous experience of participants)

Helpers will receive a structured orientation in based on the WHO PFA: Facilitator’s Manual for Orienting Field Workers. The standard training consists of a half-day orientation (approximately four hours, excluding breaks) designed to prepare helpers to support individuals recently affected by highly stressful or crisis events. Where feasible, the orientation may be extended to a full-day format (approximately five and a half hours, excluding breaks) to allow for deeper learning, skills practice, and reflection.

The training is adapted to the local cultural, linguistic, and contextual characteristics of the target population, as well as to the types of crisis situations in which helpers are likely to operate. When training is delivered with the support of interpreters, additional time is allocated, and interpreters are familiarized in advance with PFA concepts and materials to ensure fidelity and clarity.

The orientation aims to build helpers’ capacity to: (1) approach crisis situations safely for themselves and others; (2) provide practical, supportive, and humane assistance to people experiencing acute distress; and (3) avoid actions that may inadvertently cause further harm. Emphasis is placed on understanding the scope and limits of PFA, including recognition of when referral to more specialized mental health or psychosocial services is required. The training does not aim to provide clinical counselling skills.

The orientation uses participatory methods and includes opportunities for skills development and practice.

The trainings (Sh+, DWM, PFA) will be delivered by researchers and/or mental health-care professionals. All the facilitators and helpers will be additionally trained in providing support using the stress management guide (i.e., practice using the stress management in role-plays with other helpers, active supervision sessions).

**Figure 1.** Structure of the training of trainers and training of facilitators / helpers

**Outline booster session**

1. **Greeting and outlining the reason for the call (Motivation for the future and planning to continue using the skills) [3 minutes].**

**For example, say:**

“Hello {{name of participant}}, this is {{name of facilitator}} from {{name organisation}}.

I am calling you today to give you some support on using the skills you learnt with [Doing What Matters in Times of Stress (DWM)/Self-Help Plus (SH+)], it’s a chance for us to discuss how you’ve been getting on since ending the intervention and review how you’ve been getting on with using what you learnt.

Do you have 20 minutes now to talk? If not, when would be a good time for me to call you back?

One thing we know about DWM/SH+ is that, like exercising to get stronger, it’s important to continue practising the skills to handle stress better. So, this call is to help you put into practice what you have learnt.

Firstly (1), we’ll spend a few minutes seeing how you’ve been since finishing DWM/SH+, how you’ve got on with using what you learnt and how you feel when using DWM/SH+. We will then review (2) any DWM/SH+ content. As we do this, I’ll note important points as we talk and can send these to you as a plan (3) for how you might continue using DWM/SH+.

So, we are going to look at three areas:

1. How you’ve used or not used DWM/SH+ and how you feel when using DWM/SH+
2. Review of the DWM/SH+ content
3. How you might use DWM/SH+ going forward [finalising the plan]

How does that sound to you? Do you have any questions or comments before we start?”

2. **Review progress with using DMW / SH+ since finishing the intervention [8 minutes].**

*Facilitator: note down anything that is needed for the motivation plan, especially Motivation for the future and planning to continue using the techniques.*

**For example, say:**

“I’d like us now to spend a few minutes just going through how you’ve got on with using what you learnt in DWM/SH+ since the end of the sessions.”

**General questions to understand the participant’s use of the skills** [Please note that the following questions are just reference points and not all need to be addressed. Feel free to focus on the most relevant topics]:

- “Have you been able to use the DWM/SH+ skills since the end of the sessions?”
- Can you think of a specific situation where using DWM/SH+ helped you since the end of the sessions?
- “What have you used, and what has been most helpful?”
- “What’s helped you to use them?”
- “How did you feel when using the skills vs not using them?”
  *Facilitator: try here to help the participant notice any improvements in using the skills, e.g*.
  - “OK, so it seems you felt less stressed when using DWM/SH+. What do you think would happen if you used the skills more regularly?
  - “So it sounds like you didn’t benefit much from the content. Have you noticed anything, even the smallest change, due to completing DWM / SH+?”

**Questions specifically related to values and impact on relationships:**

“As you might remember, DWM/SH+ includes skills and ways that we can live by our values. I would like to take a few minutes to understand how DWM/SH+ may have impacted your relationships and your values.”

*Facilitator: if the participant has not practised the skills in the past couple of weeks/since DWM/SH+ ended, ask the participant to think back to the time the participant was enrolled in the intervention.*

- “Did using DWM/SH+ impact on any relationships in your life?”
- “What relationships and what was the impact? what difference did it make? This might be a very small change or a larger change.”
- “Did you identify any values to live by during the course?” [if no, move to next part]
- “Do you remember what values you identified you wanted to live by?”
- “Has thinking about your values and trying to live according to them impacted you and/or others around you?”
  - “What difference did it make?”
  - “What difference might it make?”

**If the participant DID PRACTICE skills/exercises – for example, say:**

“It is great that you’ve managed to find time to use the skills since the end of the intervention.

From what you’ve said, examples of where DWM/SH+ have helped you are: {{say back a summary of where DWM/SH+ has helped the participant}}.

Is that correct? Is there anything you would like to change? Or are there any skills you would like to practice more often? I will write these down on your plan.”

**If the participant DID NOT practice the skills/exercises or get any benefit – for example, say:**

“Finding time to do the exercises or use the skills can be hard. So, don’t worry if you haven’t been able to use them, let’s see if we can find anything that might help you to practice the skills in a way that is helpful for you.

- When you did DWM/SH+, how did the skills help you manage your stress?
- Was there a particular skill that you liked or helped you?
- What are the advantages of practising the skills more? What are the disadvantages?
- What difficulties have resulted from your stress and how might DWM/SH+ help you to manage this better?
- What do you think you can do to make it more likely that you will practice the skills more in the coming weeks?
- Can we find 5 minutes twice a day over the next few weeks where you can practise grounding or unhooking?
- Can you identify 2 or 3 important values that you can live by, even in very small ways, over the next few weeks?
- What were some of the problems that got in the way for you?
- Do you have any ideas on overcoming these problems or barriers?”

**Summarize the action plan – for example, say:**

“I’ll now read back to you what I’ve heard about the advantages of using DWM/SH+ and how you might do this over the coming weeks: {{say back a summary of what has been said}}.

Is that correct? Is there anything you would like to change? I will write these down on your plan.”

**If the participant does not have any ideas on how to overcome barriers – for example, say:**

“Some people I’ve spoken with have found that setting a time to do their activity in advance or explaining to their family that they need some time to do the intervention helps. Would either of these be something you can try?”

3. **Practicing skills/exercises [2-5 minutes].**

**For example, say:**

“Let’s briefly go over the five different tools that are in DWM/SH+. These are:

- Grounding
- Unhooking
- Acting on your values
- Being Kind
- Making Room

Remember you can find descriptions of all of these in the tools section {{back of book or in the app}}.

- Would you like to review any of these tools on the call with me?
  *Facilitator: if the answer is YES, use the existing scripts in the DWM manual.*
- Are there any other tools from DWM/SH+ that you would like to try using or that you will review?”

**Offer to do a grounding exercise with the participant and ask how they felt afterwards.**

4. **Finalising the plan [3 minutes].**

**For example, say:**

“Thanks so much for your time today. We’ve covered a lot about how you will use DWM/SH+ going forward. I just want to read back to you what I’ve written down, I’ll send this to you after the call.”

**Summarise what the person has said, write this up, and offer to send it to the participant.**

**While reading back to the participant, divide the notes into the following areas:**

**Examples of how DWM/SH+ have helped me:**

- *It made me feel less stressed generally each day.*
- *I was able to focus more on my family.*
- *It did not help me, as I’ve not managed to use what I learnt / it just hasn’t been effective.*
- *Identifying my values of X,Y,Z has helped me feel less stressed. I feel better living by these.*

**Examples of how I feel when using DWM/SH+:**

- *It helps me feel more calm and focused.*
- *I feel more able to deal with my life and with stress.*

**Examples of how I plan to use DWM / SH+ going forward:**

- *I will practise grounding and unhooking when I brush my teeth each day*
- *I will try to live by my values of {{X, Y and Z}} by {{XXX}}.*

**For example, say:**

“Is that correct? Is there anything you would like to change?

OK, great, we now have a plan, which I will send to you after this call.”

5. **Finish the call [1 minute].**

**For example, say:**

“We have a few minutes left to talk. Do you have any questions, or is there anything else you would like to say before I go?”

NOTE:

*If a participant says they are overwhelmed by problems* *and are asking for your help, then consider taking a few minutes to help identify support and coping strategies (see* section 7.1 *on support coping and* section 7.2 *on participants experiencing distress in the DWM manual).*

**Outline psychological placebo**

**Session 1 (WELCOME AND FIRST AREA)**

6. **Greeting and outline reason for the call (5 minutes)**

*“Hello [name of the person], this is [name of the facilitator] from the University of Verona / Roma. [Recruiter’s name] gave me your phone number. Do you remember? Probably [Recruiter’s name] told you that I would call you as part of the ADVANCE project. Is it a good moment? This call is a chance for me to explain you my role, for us to discuss about some topics that I will explain you in a moment, and for you to ask any questions you may have.*

*My role as a helper is to offer support, giving you information on existing local services that can be useful to you or your family and friends. I am not a professional psychotherapist, but I am here to guide you toward local resources that may be beneficial in addressing your needs, whether they relate to health, well-being, or any other area where professional services might be necessary. By providing this information, my aim is to make it easier for you to navigate the options available and find the support that best meets your needs.*

*I will contact you for a maximum of 15/20 minutes per week, for a total of five weeks, with followed by a follow up call one month after the fifth call.*

*In each call, we are going to look at one of these topic areas:*

- *Education*
- *Health and social services*
- *Legal issues, migration*
- *Social activities, support for families*
- *Housing*
- *Work*

*At the end of each call, we will schedule the date and time for the next appointment. I will send you a reminder that we have a meeting the day before.*

*Feel free to contact me if you need to rearrange a meeting [Please confirm which contact details you will provide to your participants: private phone number, email, project phone number, or project email?]. I do not monitor my phone or messages outside work hours, so you should not expect a response during these times. If you have any urgent need for support, you should call or go to the nearest emergency healthcare facility.*

*Do you have any questions or is there anything else you would like to say before we start with the first area?”*

7. **Review of the first area (5 to 10 minutes)**

[The order in which services will be described is flexible, and it could be adapted to the specific needs of participants.]

**[If the recruiter told you the first topic chosen by the participant]**

*“I know that [recruiter's name] had already mentioned the structure of the calls to you, and that you were interested in talking about [name of the area chosen by the participant] together.”*

**[If the recruiter did not tell you the first topic chosen by the participant]**

*“Is there a specific area you’d like to focus on today? We can start with the area that interests you the most.*

*Let's start with the [area’s name]”*

[You will start to explain all the services related to the chosen area - see appendixes]

**[If the participant has been in Italy for a long time and tell you that he/she knows the Verona/Rome services well, you could say:]**

*"I see you are familiar with these services. Have you ever helped a friend or family member with this? Could they benefit from these services? Just so you know, they are available..“* [Continue with the description of the services (see appendices)].

*or*

*"I see you are familiar with these services. Was it difficult for you to (example: learn Italian? To get the documents? To make friends? To find a job? To find a place to live?) Because for many non-Italian people, it can be quite complex.”*

**[If it seems they are overwhelmed by the problems and are asking for your help**, then consider suggesting other local organization or resources (e.g. community leader, refugee organizations, friends, family doctor) that may be helpful to the person. Encourage participants to connect with these other resources, but keep in mind that it is the person’s decision whether they will follow through with these suggestions.]

8. **Finish the call (1 minute)**

*“We have a few minutes left to talk. Do you have any questions or is there anything else you would like to say before we finish our conversation? Next week, what area would you like to explore further? Remember that today we covered [the chosen topic], and the following areas left are [name of the other areas].”*

[If the person was overwhelmed by the problems remind again what you discussed/described before.]

*“Thank you [name], would it be OK for you to call you on [date and time]?*

*OK, I will call again next week on the [date and time].”*

**Generic support call (session 2, 3, 4, 5)**

1. **Greeting (1 minute):**

*“Hello [name of the person], this is [name of the facilitator] from the University of Verona / Roma. How are you? Is it a good moment?*

*Today, as planned, we’ll discuss the [name of area]. Would be that OK for you?”*

2. **Ask the participant how they have been since the last call, what their plans are, and activities for the next week (4 minutes)**

*“Before we start with the second/third/fourth/fifth area, we can spend a few minutes discussing how your week was.* [Please note that the following questions are just reference points and not all need to be addressed. Feel free to focus on the most relevant topics]

- *Was there anything that you found helpful based on what I shared with you last week?*
- *Was there anything that you found confusing? Is there something you would like to clarify?*
- *Do you have any plans for next week based on what we've already discussed?”*

**[This may lead to the participant talking about other problems they have faced, also in other areas**. If this happens that is ok, allow them to talk and express themselves and show empathy (*examples: “I see that this is difficult/ I see that it is a complex situation/ It would be difficult for you to manage a situation like this….”).* Remind that you are not a professional psychotherapist, but you are here to guide him/her toward local resources that may be beneficial in addressing his/her needs, whether they relate to health, well-being, or any other area where professional services might be necessary.

After giving some space and showing empathy, gently return to the topic. Remember, you only have 15 minutes for the call.]

**[If it seems they are overwhelmed by the problems and are asking for your help**, then consider suggesting other local organization or resources (e.g. community leader, refugee organizations, friends, family doctor) that may be helpful to the person. Encourage participants to connect with these other resources, but keep in mind that it is the person’s decision whether they will follow through with these suggestions.]

3. **Review of the second/third/fourth/fifth area (5 to 10 minutes).**

[The order in which services will be described is flexible, and it could be adapted to the specific needs of participants.]

*“Let's start with the [area’s name]”*

[You will start to explain all the services related to the chosen area - see appendixes*].*

**[If the participant has been in Italy for a long time and tell you that he/she knows the Verona/Rome services well, you could say:]**

*"I see you are familiar with these services. Have you ever helped a friend or family member with this? Could they benefit from these services? Just so you know, they are available...”* [Continue with the description of the services (see appendices)].

*or*

*"I see you are familiar with these services. Was it difficult for you to (example: learn Italian? To get the documents? To make friends? To find a job? To find a place to live?) Because for many non-Italian people, it can be quite complex.”*

**[If it seems they are overwhelmed by the problems and are asking for your help**, then consider suggesting other local organization or resources (e.g. community leader, refugee organizations, friends, family doctor) that may be helpful to the person. Encourage participants to connect with these other resources, but keep in mind that it is the person’s decision whether they will follow through with these suggestions.]

4. **Finish the call (1 minute)**

*“We have a few minutes left to talk. Do you have any questions or is there anything else you would like to say before we finish our conversation? Next week, what area would you like to explore further? Remember that today we covered [the chosen topic], and the following areas left are [name of the other areas].”*

[If the person was overwhelmed by the problems remind again what you discussed/described before.]

[For session 2, 3, 4:]

*“Would it be OK for you to call you on [date and time]?*

*OK, I will call again next week on the [date and time].”*

[For session 5:]

*“Thank you so much for your time. Over these five weeks, we’ve covered a lot about local and national services in [Verona / Roma], and I hope it has been helpful. I will call you in a month to check in on how things are going and to summarize everything.”*

*“Would it be OK to call you on [date and time]? OK, I will call next month on the [date and time].”*

**Additional call (session 6)**

1. **Greeting and outline reason for the call (1 minute)**

*“Hello [name of the person], is [name of the facilitator] from the University of Verona /Rome. How are you?*

*I am calling you today to give you an overview of the topic discussed during the last month. It’s also a chance for us to discuss and to help with anything you found helpful or difficult.”*

2. **Ask the participant how they have been since the last call, and what are their plans and activities for the future (5 - 10 minutes)**

*“Before we start with the overview of the five areas, we can spend a few minutes discussing how this month was. [Please note that the following questions are just reference points and not all need to be addressed. Feel free to focus on the most relevant topics]*

- *Was there anything in particular that you found helpful based on what I shared with you last month?*
- *Was there anything that you found confusing? Is there something you would like to clarify?*
- *Do you have any plans for the future based on what we've already discussed?”*

**[If the participant doesn’t remember your discussions, you could say based on your notes:]**

*"I remember that during one of our calls, you mentioned that you wanted to (examples: find a job/ wanted to start a course/ wanted to see a psychologist). How did that go?"*

[If needed, summarize the services available for that area (see appendices)]

**[If the participant wasn’t interested in any of the five areas:]**

*"I remember you told me that you were happy with your job/ you were satisfied with your doctor/ you had just gotten your citizenship, right? Do you still feel that way? How’s it going?"*

***[This may lead to the participant talking about other problems they have faced, also in other areas****.* If this happens that is ok, allow them to talk and express themselves and show empathy (*examples: “I see that this is difficult/ I see that it is a complex situation/ It would be difficult for you to manage a situation like this….).* Remind that you are not a professional psychotherapist, but you are here to guide him/her toward local resources that may be beneficial in addressing his/her needs, whether they relate to health, well-being, or any other area where professional services might be necessary.]

[After giving some space and showing empathy, gently return to the topic. Remember, you only have 15 minutes for the call.]

**[If it seems they are overwhelmed by the problems and are asking for your help**, then consider suggesting other local organization or resources (e.g. community leader, refugee organizations, friends, family doctor) that may be helpful to the person. Encourage participants to connect with these other resources, but keep in mind that it is the person’s decision whether they will follow through with these suggestions.]

3. **Finish the call (1 minute)**

*“We have a few minutes left to talk. Do you have any questions or is there anything else you would like to say before we finish our conversation?”*

[If the person was overwhelmed by the problems remind again what you discussed/described before.]

*“Thank you so much for the time you dedicated to us today. We covered a lot about local and national services during this program, and I hope it was helpful. Good luck!”*

**7. Referral pathway for study participants in need of structured clinical support**

Participants requiring mental health care beyond the low-intensity interventions and/or psychological placebo provided within the context of this trial will be referred to appropriate clinical services according to a predefined referral pathway. Clinical needs may emerge in any phase of the trial, such as during screening, intervention delivery, booster sessions, follow-up assessments, or through spontaneous disclosure. Referral criteria include severe depressive symptoms, suspected psychotic disorders or severe substance use or alcohol use disorders, acute psychological deterioration, safeguarding concerns, or any other condition requiring specialist assessment based on clinical judgement. Research staff and peer helpers will be trained to recognize these situations and will immediately notify the site clinical supervisor (clinical psychologist or psychiatrist). The clinical supervisor will assess the participant and activate the appropriate referral pathway, including: (1) local emergency psychiatric services for participants at immediate risk; (2) local Community Mental Health Services (CSM); (3) public or community psychological services for non-urgent mental health needs; and (4) social or protection services when psychosocial needs predominate. Where participants consent, their General Practitioner may also be informed to facilitate continuity of care and communication.

Referral to clinical services will not automatically lead to study withdrawal, because clinical services can integrate low-intensity psychosocial interventions. Participants may continue to attend the intervention’s session, study assessments whenever clinically appropriate, and any concomitant mental health treatment initiated during follow-up will be documented. All referrals and related actions will be recorded in study documentation and managed in accordance with Good Clinical Practice (GCP), GDPR, and local ethical requirements.

**8. Description of the implementation measures**

Implementation outcomes have been selected and will be interpreted according to Proctor et al.'s Implementation Outcomes Framework. The study will assess:

**Losses to follow-up:**

Losses to follow-up refer to participants who do not complete outcome assessments by the end of the study and participants who withdraw their consent during the study. The number of losses to follow-up and the reasons for attrition at T2 and T3 will be documented using ad hoc forms.

**Fidelity to the intervention manual:**

Intervention fidelity will be assessed to determine the extent to which the intervention is delivered by facilitators, and received by participants as intended, and to identify any deviations from the WHO intervention manual. Fidelity assessment will consider adherence to the prescribed content and procedures, the intervention dose delivered, the quality of delivery, and participants’ exposure and responsiveness. The research team will complete ad-hoc WHO fidelity forms. In addition, for DWM, we plan to use DWM metadata to track participants' access to the intervention sessions.

#### Intervention acceptability

From an implementation perspective, acceptability refers to participants’ perception that the intervention is agreeable, satisfactory, useful/important, or appropriate, based on their cognitive and emotional reactions and responses to it. Acceptability is a multidimensional and potentially dynamic construct that may change across people and over time, as participants become familiar with the intervention. In this study, acceptability will therefore be assessed using both participants’ self-reported perceptions and behavioural indicators of engagement with the intervention. Specifically, acceptability will be evaluated by collecting: 1) adverse events as reported by participants at T2 and T3; 2) the number of sessions accessed for each condition at T2 and T3; 3) scores on the Acceptability of Intervention Measure (AIM) subscale at T2 and T3. Adverse events are defined as any undesirable experience occurring to a subject during the study, whether considered related to the trial procedure or the SH+ / DWM intervention. All adverse events reported spontaneously by the subject or observed by the investigator or staff will be recorded for ethical reasons and as a proxy measure of the intervention's acceptability.Number of sessions accessed for each condition: SH+ (number of group sessions attended), DWM (number of modules unlocked and number of helper calls) and psychological placebo (number of helper calls). The Acceptability of Intervention Measure (AIM) is a subscale of 4 items, 5-point Likert scale: 1 = completely disagree to 5 = completely agree)45. The unit of measure is a descriptive scale rating the level of acceptability of the intervention.

#### Intervention appropriateness

From an implementation perspective, appropriateness refers to the perceived fit, relevance, or compatibility of an intervention for a specific population, problem, setting, or service-delivery modality and context. It concerns whether participants consider the intervention appropriate and suitable for addressing the identified needs within the specific circumstances in which it is delivered. Although related to acceptability, appropriateness is a distinct construct: an intervention may be perceived as agreeable or satisfactory but not necessarily relevant or well suited to a particular population or context. Perceptions of appropriateness may also change over time as participants gain familiarity, and direct experience with the intervention. In this RCT, appropriateness will be assessed at T2 and T3 with the "Intervention Appropriateness Measure" (IAM) subscale (4 item, 5-point Likert scale: 1 = completely disagree to 5 = completely agree)45. The unit of measure is a descriptive scale that rates the appropriateness of the intervention.

#### Intervention feasibility

From an implementation perspective, feasibility refers to the extent to which an intervention can be successfully delivered or carried out within a specific population, context, or service-delivery setting. It reflects perceptions of whether the intervention is practicable and manageable, considering factors such as the time required for training, delivery, and supervision, effort, resources (including practical resources), procedures, and support required for participation. Feasibility is related to but distinct from acceptability and appropriateness: for example and in many contexts, an intervention may be perceived as satisfactory and relevant, while still being difficult to implement or use in practice, for different reasons. Perceived feasibility may also change over time as participants gain direct experience with the intervention and its delivery procedures.

Feasibility will be assessed at T2 and T3 with the "Feasibility of Intervention Measure" (FIM) subscale (4 items, 5-point Likert scale: 1 = completely disagree to 5 = completely agree)45. The unit of measure is a descriptive scale rating the level of feasibility of the intervention.
